# Rehabilitation effects of circuit resistance training in coronary heart disease patients: A systematic review and meta‐analysis

**DOI:** 10.1002/clc.23855

**Published:** 2022-06-27

**Authors:** Chunchun Wu, Rongsheng Bu, Yaoguo Wang, Chaoxiang Xu, Youfang Chen, Lishuang Che, Shengnan Wang

**Affiliations:** ^1^ Department of Cardiology The Second Affiliated Hospital of Fujian Medical University Quanzhou Fujian China; ^2^ Department of Clinical Medicine Quanzhou Medical College Quanzhou Fujian China

**Keywords:** aerobic training, circuit resistance training, coronary heart disease, meta‐analysis, peak oxygen uptake, randomized controlled trial

## Abstract

**Background and Hypothesis:**

The rehabilitation effect of circuit resistance training in coronary heart disease (CHD) patients remains unclear. We perform this review to examine the rehabilitation effect of circuit resistance training in CHD patients and to provide a basis for the formulation of reasonable individual exercise prescriptions for CHD patients.

**Methods:**

Randomized controlled trials (RCTs) were searched on PubMed, Web of Science, The Cochrane Library, Embase, Clinical Trials, and CNKI. About 1232 studies were identified. Nine RCTs were finally used for the present meta‐analysis to determine the rehabilitation effect of circuit resistance training in CHD patients, compared to aerobic training. Individuals enrolled for the studies were at a mean age of 60.5 years old and were all CHD patients. Following the PRISMA guidelines, we extracted basic information about the study and patient characteristics, as well as measurements (e.g., the peak oxygen uptake, the body mass index [BMI], the body fat percentage, the systolic blood pressure, the total cholesterol, and triglycerides). Subsequently, this meta‐analysis determined the overall effect by using standardized mean difference (SMD) and 95% confidence interval (CI).

**Results:**

Compared with aerobic training, circuit resistance training significantly decrease the BMI and the body fat percentage.

**Conclusions:**

As suggested from the present meta‐analysis of RCTs, circuit resistance training is effective in improving the BMI and the body fat percentage in CHD patients and may help delay the progression of CHD. CRT has the advantage of lower load in most cases with a similar effect.

## 1 INTRODUCTION

Coronary heart disease (CHD) refers to one of the most common cardiovascular system diseases, which has imposed a huge social, medical, and economic burden.^1^ Obesity and lack of exercise were believed as independent factors affecting the development of CHD.^2^ Regular physical activity and systematic exercise are vital components of most cardiovascular disease treatments, which are associated with the all‐cause mortality of the cardiovascular disease. Accordingly, some guidelines^3^ have formulated the cardiac rehabilitation strategies based on exercise therapy as an attempt to help patients reasonably avoid cardiovascular disease‐related risk factors and positively facilitate disease prevention and treatment.

Cardiorespiratory Fitness (CRF) refers to an effective and independent predictor of cardiovascular mortality and the risk of all‐cause mortality. It is recommended as a clinical indicator, in accordance with a statement issued by the AHA.^4^ Exercise training is capable of significantly improving the CRF of patients with CHD, improving the quality of life and long‐term prognosis of patients, and greatly reducing the mortality of CHD.^5^

Over the past few years, some scholars have proposed circuit resistance training, inconsistent with conventional simple resistance training, which is progressive resistance training based on aerobic training. Compared with conventional resistance training, it is characterized by lighter weight, more repetitions, and shorter training intervals.

Exercise test assessment has been the most extensively used functional test, which can provide a relatively scientific and reasonable basis for formulating exercise prescription.^6^ And anaerobic threshold level acts as a vital reference index for the formulation of exercise prescriptions for CHD patients. Resistance exercise can help to increase myocardial perfusion and improves myocardial ischemia while improving muscle mass and exercise ability.^7^

As indicated from existing studies, aerobic training combined with resistance exercise and aerobic training can significantly improve the cardiopulmonary response in patients with CHD.^7^ Hansen et al.'s study^8^ reported that aerobic combined with resistance exercise exerts a better effect on exercise therapy for patients with CHD than aerobic training independently. However, controversies remain about the evidence of whether circular resistance training can improve cardiopulmonary endurance, weight loss, and lipid reduction in CHD patients.

This systematic review aimed to examine the exercise training effect of circuit resistance training in CHD patients and lay a basis for the formulation of reasonable individual exercise prescriptions for CHD patients.

### 1.1 Article type

This study is a systematic review and meta‐analysis.

## 2 MATERIALS AND METHODS

### 2.1 PRISMA statement

The present meta‐analysis was conducted following the recommendations of the PRISMA statement.^9^

### 2.2 Literature search

PubMed, Web of Science, The Cochrane Library, Embase, Clinical Trials, and CNKI were comprehensively searched by two independent reviewers (Chunchun Wu and Rongsheng Bu) on October 21, 2021, without any language or date restrictions. Relevant studies were identified for CRT versus Standard resistance training on improving fitness. And the studies identified included random controlled trials and observational studies.

The search terms for CHD are presented below:(Coronary Disease"[Mesh]) OR (Coronary Diseases) OR (Disease, Coronary) OR (Coronary Heart Disease) OR (Disease, Coronary Heart) OR (Heart Disease, Coronary) OR (coronary heart disease) OR (angina) OR (myocardial infarction) OR (ischemic heart disease) OR (coronary artery bypass). The search terms for circuit resistance training are presented below: (circuit resistance training) OR (Circulating resistance training) OR (Combined Exercise Training) OR (resistance training) OR (aerobic‐resistance exercises) OR (weight training) OR (muscle strengthening) OR (progressive resistance training) OR (circuit training) OR (exercise training). The search terms for the healing effects function are presented below: (physical function) OR (aerobic capacity) OR (exercise capacity) OR (exercise tolerance) OR (VO_2_ peak).

Two of us (Chunchun Wu and Rongsheng Bu) searched the database based on the previously specified search terms and search strategies and filtered the studies independently. Any disagreement was reported to the third reviewer (Shengnan Wang) and up to him to decide. And we excluded those studies that not met our inclusion criteria.

### 2.3 Inclusion and exclusion criteria

Inclusion criteria included the following: stable angina pectoris; old myocardial infarction; percutaneous transluminal coronary angioplasty; and coronary artery bypass grafting. The training period should be no less than 7 days. Studies should report the rehabilitation effect of circuit resistance training in CHD patients compared to aerobic training. The outcome measures should include at least one of these: the peak oxygen uptake, the body mass index (BMI), the body fat percentage, the systolic blood pressure, the total cholesterol, and triglycerides (TGs).

Exclusion criteria consisted of: the presence of uncontrollable arrhythmias; unstable angina pectoris, and uncontrolled high blood pressure; congestive heart failure, exercise‐induced angina; exercise‐induced hypotension; and subjects with absolute exercise restriction. Review articles, case‐reports were not used. Studies with incomplete follow‐up work were excluded.

### 2.4 Data extraction

All eligible studies were identified by two authors, and the differences were resolved by reaching a consensus with a third researcher.

Several data were extracted: (e.g., the first author article, the country of publication, the year, the number of patients, the average age, the duration of exercise intervention, the peak oxygen uptake, the BMI, the body fat percentage, TG, the total cholesterol, and the systolic blood pressure).

### 2.5 Statistical analysis

RevMan V. 5.2 and STATA 15.0 were used for statistical analysis. Combined SMD and its 95% confidence interval [CI] were measured. *I* ^2^ test was calculated as a measure of heterogeneity, for which we believe *I* ^2^ values of 25%, 50%, and 75% indicate a low, moderate, or high heterogeneity.^10^ We took the heterogeneity into account during the evaluation of the statistical effect. And the quality of the studies was assessed independently by two of us (Chunchun Wu and Yaoguo Wang). The random‐effects model was adopted to combine effect size when *I* ^2^ > 50%; otherwise, we used a fixed‐effects model. The Begg test was performed to assess publication bias. Finally, a two‐tailed *p *< .05 was considered statistically significant.

## 3 RESULTS

### 3.1 Searching results

**Figure 1:**
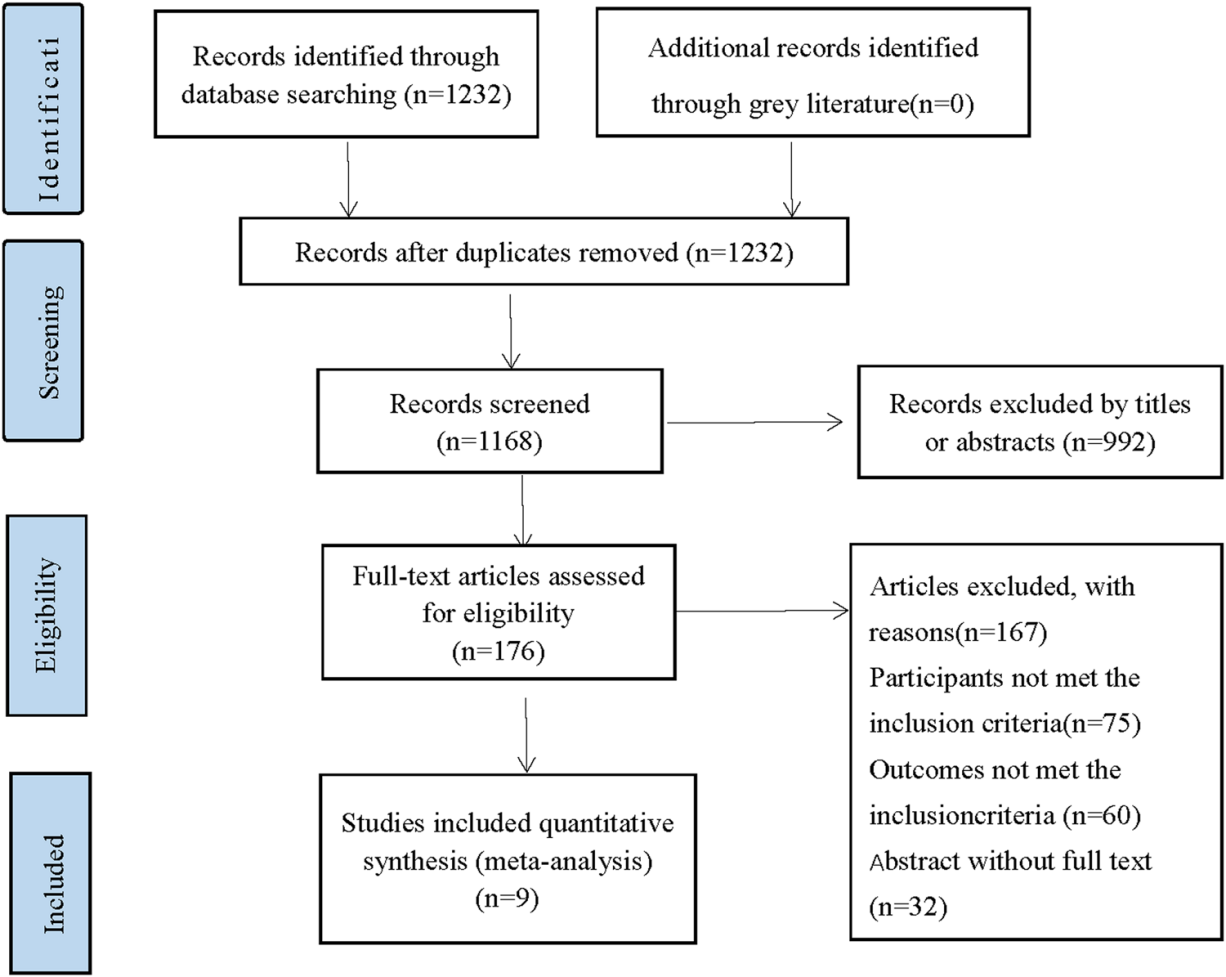
PRISMA flow chart of literature identifying

After eliminating duplicates, our search initially identified a total of 1232 references. Next, 176 articles were deemed eligible after a review of titles and abstracts. Then, for the reasons listed in Figure 1, 167 studies were further removed. And we have found some incomplete studies by searching the registry center. Unfortunately, we were unable to contact the relevant units or authors for relevant data. Finally, nine randomized controlled trials (RCTs) focused on the rehabilitation effect of circuit resistance training were used in the meta‐analysis.^7^, ^11^, ^12^, ^13^, ^14^, ^15^, ^16^, ^17^, ^18^ And those in the intervention groups were trained by circuit resistance training, while those in the control groups were trained by aerobic training. We outlined the retrieval strategy in Figure 1.

### 3.2 Study characteristics

Nine RCTs were included in the meta‐analysis.

Basic information from publications, mean age, exercise program, main outcome indicators, duration of exercise, and exercise frequency is listed in Table 1. And to show the effect of different training methods on the outcomes more intuitively, we define a new variable: the time ratio, which means the product of the training period and the training duration. And all the absolute values were divided by the time ratio to get the outcomes adjusted by training intensity. The adjusted outcomes were summarized in Table 2.

**Table 1:** Characteristics of studies

Study	Year	Country	Age (years)	Sex ratio (male%)	Circuit resistance training（*n*）	Aerobic training（*n*）	Outcomes	Duration (months)	Frequency (times per week)	Dropouts (*n*)	Adverse events	Beta‐blockers use (%)
Theodorou^11^	2016	Greece	AT:age = 61 ± 7; CT:age = 64 ± 6	100	15	15	②③④⑤	8	3	0	0	NA
Santa‐Clara^12^	2003	Portugal	AT:age = 57 ± 11; CT:age = 55 ± 10	100	13	13	②③	12	3	0	0	NA
Vona^13^	2009	Switzerland	AT:age = 56 ± 6; CT:age = 55 ± 9	75.5	53	52	①②④⑤⑥	1	4	0	0	NA
Hansen^14^	2011	Belgium	AT:age = 58.9 ± 7.2; CT:age = 60.4 ± 8.9	95	22	25	①④⑤	1.6	3	13	1	72.3
Tofas^15^	2021	Greece	AT:age = 61 ± 7; CT:age = 64 ± 6	NA	15	15	⑥	8	3	0	0	NA
Marzolini‐1^16^	2008	Canada	AT:age = 57.9 ± 2.6; CT:age = 60.9 ± 2.3	11.3	18	14	①	6	5	13	4	83
Marzolini‐2^16^	2008	Canada	AT:age = 57.9 ± 2.6; CT:age = 62.7 ± 2.7	11.3	18	14	①	6	5	13	4	83
Santa‐Clara^17^	2002	Portugal	AT:age = 57 ± 11; CT:age = 55 ± 10	100	14	14	①	12	3	0	0	50
Gayda^18^	2009	France	AT:age = 55 ± 8; CT:age = 55 ± 8	100	8	8	①③	1.75	3	0	0	68.8
Lepretre^7^	2016	France	AT:age = 64.6 ± 9.1; CT:age = 63.1 ± 7.0	100	16	16	①	1	5	0	0	100

*Note*: ① VO_2_ peak; ② BMI; ③ Fat mass (FM, in %); ④ TG; ⑤ CHO; ⑥ SBP; ⑦ AT: aerobic training; ⑧ CT, circuit resistance training; ⑨ NA, Not available.

**Table 2:** Standardized outcomes according to training intensity

	Outcomes														
	VO_2_		BMI		Fat mass		SBP		CHO		TG		Duration	Frequency	Time ratio
Study	Interventional	Control	Interventional	Control	Interventional	Control	Interventional	Control	Interventional	Control	Interventional	Control	(Month)	(times per week)	
Theodorou‐1^11^	NA	NA	1.2	1.3	1.4	1.4	NA	NA	0.2	0.2	0.1	0.1	8	3	24
Theodorou‐2^11^	NA	NA	1.2	1.3	1.3	1.3	NA	NA	0.2	0.2	0.0	0.0	8	3	24
Santa‐Clara^12^	NA	NA	0.7	0.8	0.7	0.9	NA	NA	NA	NA	NA	NA	12	3	36
Vona^13^	6.6	6.4	6.5	6.8	0.0	0.0	46.5	45.5	39.5	40.8	37.8	38.5	1	4	4
Hansen^14^	424.6	435.2	NA	NA	NA	NA	NA	NA	29.0	29.8	21.0	24.4	1.6	3	4.8
Tofas‐1^15^	NA	NA	NA	NA	NA	NA	5.4	5.3	NA	NA	NA	NA	8	3	24
Tofas‐2^15^	NA	NA	NA	NA	NA	NA	5.1	5.3	NA	NA	NA	NA	8	3	24
Marzolini ‐1^16^	0.8	0.8	NA	NA	NA	NA	NA	NA	NA	NA	NA	NA	6	5	30
Marzolini ‐2^16^	0.7	0.8	NA	NA	NA	NA	NA	NA	NA	NA	NA	NA	6	5	30
Santa‐Clara^17^	1.2	0.9	NA	NA	NA	NA	NA	NA	NA	NA	NA	NA	12	3	36
Gayda^18^	5.9	4.6	NA	NA	5.3	5.7	NA	NA	NA	NA	NA	NA	1.75	3	5.25
Lepretre^7^	3.5	3.2	NA	NA	NA	NA	NA	NA	NA	NA	NA	NA	1	5	5

*Note*: ① VO_2_ Peak: maximum oxygen flow per minute; ② BMI: body mass index (kg/m^2^); ③ SBP: systolic blood pressure; ④ CHO: cholesterol; ⑤ TG: triglyceride; ⑥ NA: not available; ⑦ Time value: the absolute value/(duration*frequency).

### 3.3 Risk of bias

**Figure 2:**
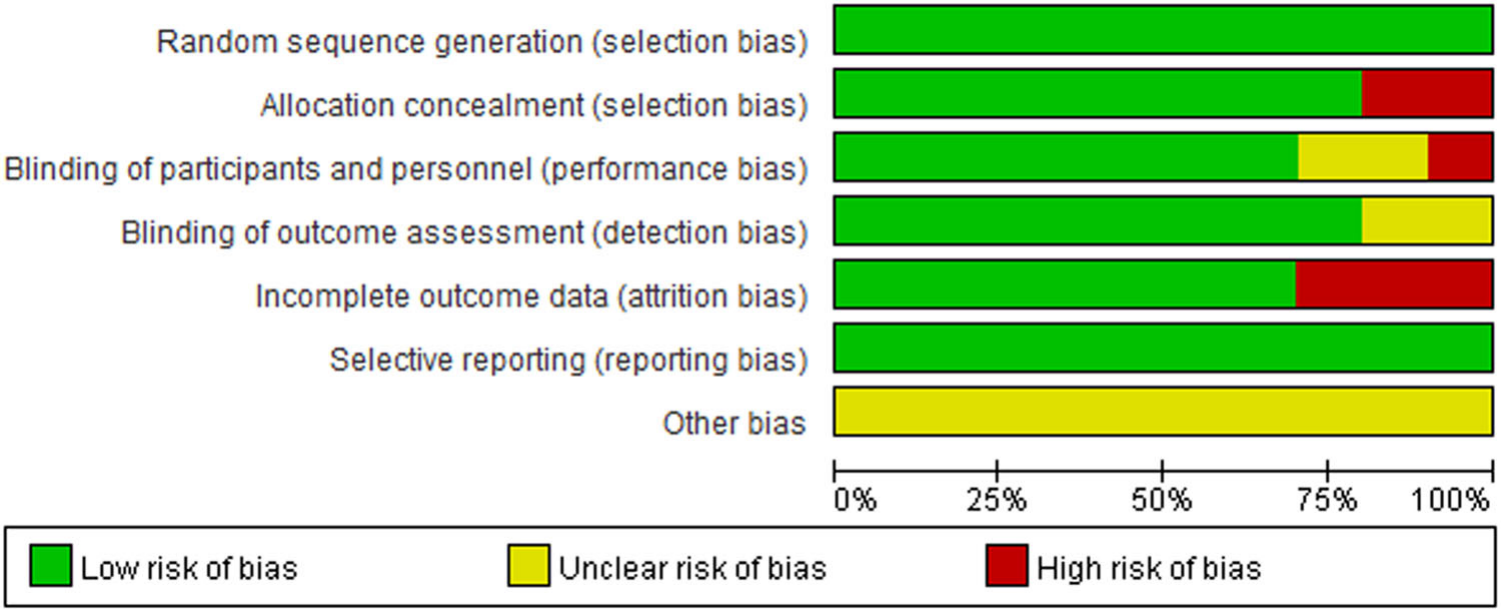
Risk bias summary

**Figure 3:**
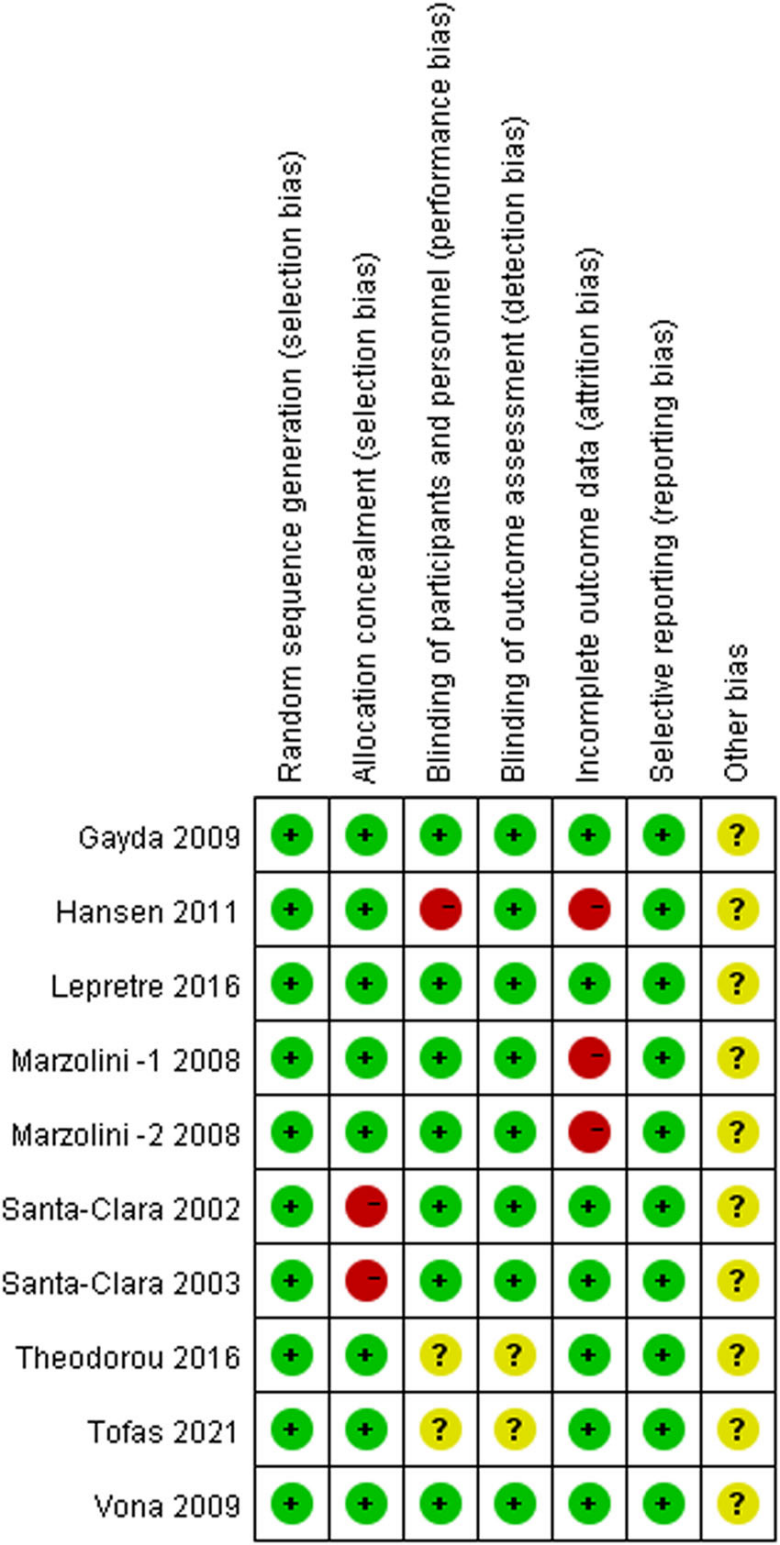
Risk biases of each study

Risk assessment of bias is shown in Figures 2 and 3, and All studies had a low‐risk bias in randomized, selective reporting. Most studies were well done for assignment hiding, participant blindness, evaluator blindness, and data integrity. It also indicated that there might be other deviation risks in the study.

### 3.4 Pooled analysis

**Figure 4:**
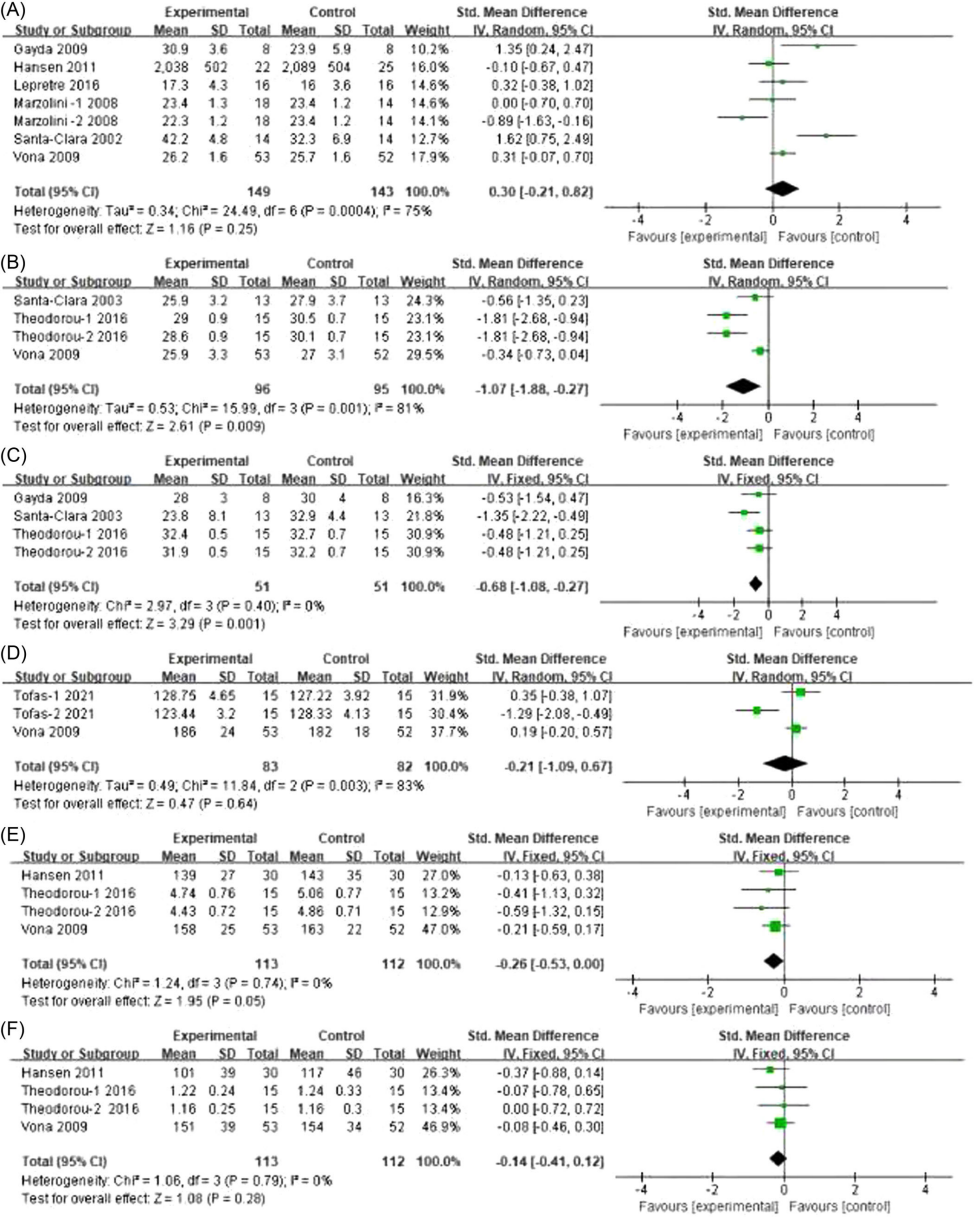
Forest plots of each outcome measure: (A), the VO_2_ peak; (b), the BMI; (C), the Fat mass (FM, in %); (D), the SBP; (E), the CHO; and (F), the TG. Block size is an inverse function of accuracy. BMI, body mass index; CHO, cholesterol; TG, triglyceride.

VO_2_ Peak heterogeneity test reported statistically significant difference (*Χ* ^2^ = 24.49, *p* = .0004; *I* ^2^ = 75%). Therefore, random effect model was selected for the further analysis and processing result display. No statistically significant difference was reported in VO_2_ peak between the experimental and the control groups (SMD = 0.30, 95% CI = −0.21 to 0.82, *z* = 1.16, *p* = .25; Figure 4).

BMI heterogeneity test showed statistically significant differences (*Χ* ^2^ = 15.99, *p* = .001; *I* ^2^ = 81%). Therefore, random effect model was selected for the further analysis and processing. result display, the BMI difference between the experimental and the control groups was statistically significant (SMD = −1.07, 95% CI = −1.88 to −0.27, *z* = 2.61, *p* = .009; Figure 4).

Heterogeneity test of the body fat percentage showed no statistical significance (*Χ* ^2^ = 2.97, *p* = .40; *I* ^2^ = 0%). Accordingly, the fixed effect model was selected for further analysis and treatment. Result display, the difference in the body fat percentage between the experimental and the control groups was statistically significant (SMD = −0.68, 95% CI = −1.08 to −0.27, *z* = 3.29, *p* = .001; Figure 4).

Systolic blood pressure heterogeneity test difference was statistically significant (*Χ* ^2^ = 11.84 *p* = .003; *I* ^2^ = 83%). Thus, random effect model was selected for the further analysis and processing. Result display, no significant difference was identified in the systolic blood pressure between the experimental and the control groups (SMD = −0.21, 95% CI = −1.09 to 0.67, *z* = 0.47, *p* = .64; Figure 4).

Heterogeneity test of the total cholesterol showed no statistical significance (*Χ* ^2^ = 1.06 *p* = .79; *I* ^2^ = 0%). Therefore, the fixed effect model was selected for the further analysis and treatment. Result display, no significant difference was identified in the total cholesterol between the experimental and the control groups (SMD = −0.26, 95% CI = −0.53 to 0.00, *z* = 1.95, *p* = .05; Figure 4).

No significant difference was identified in TG heterogeneity test (*Χ* ^2^ = 1.06 *p* = .79; *I* ^2^ = 0%). Therefore, the fixed effect model was selected for further analysis and treatment. Result display, no significant difference was identified in TG between the experimental and the control groups (SMD = −0.14, 95% CI = −0.41 to 0.12, *z* = 1.08, *p* = .28; Figure 4).

### 3.5 Publication bias

Supporting Information: Figure 1 indicates that VO_2_ Peak may have a publication bias risk. However, the Begg test found no evidence of publication bias (*p *=.652).

Supporting Information: Figure 2 shows the possibility of publication bias in the BMI. However, the Begg test found no evidence of publication bias (*p *= .09).

Supporting Information: Figure 3 shows no possibility of publication bias in the body fat percentage. The Begg test found no evidence of publication bias (*p *= .174).

Supporting Information: Figure 4 presents a possible risk of publication bias in the systolic blood pressure. The Begg test found no evidence of publication bias (*p *= .117).

Supporting Information: Figure 5 shows no possible risk of publication bias for the total cholesterol. The Begg test reported no evidence of publication bias (*p *= .174).

Supporting Information: Figure 6 shows that there is no possibility of publication bias risk for triglycerides. The Begg test found no evidence of publication bias (*p *= .497).

## 4 DISCUSSION

To the best of the authors' knowledge, this has been the first randomized controlled meta‐analysis to date to investigate the rehabilitation effects of circuit resistance training in CHD patients. As demonstrated from the results of this meta‐analysis, compared with aerobic training, circuit resistance training has insignificantly impacted the peak oxygen uptake, the systolic blood pressure, total blood cholesterol, and triglyceride in CHD patients, whereas it can significantly decrease the BMI and the body fat percentage.

The meta‐analysis of RCT shows its own advantages and the conclusions drawn are more reliable and accurate since all the data originate from RCTs. As indicated by Begg tests, no significant evidence of publication bias was found in our study. According to the results, the VO_2_ Peak, the BMI, the SBP group *I* ^2^, respectively (*I* ^2^ = 75%, *I* ^2^ = 81%, *I* ^2^ = 83%) were more than 50%, and the *p* values (*p* = .0004, *p* = .001, *p* = .003) were less than 50%. Significant heterogeneity was identified among studies. Considerations may be correlated with the following factors. I. Clinical heterogeneity: Differences were found in the gender, age, race, and comorbidity of the study population. Different exercise frequencies, intensities, durations, and methods may cause differences. A range of measurement methods and observation time points of outcome indicators may cause significant differences in effect. II. Heterogeneity of methodology. According to Figure 3, the selected studies Santa‐Clara 2002, Santa‐Clara 2003 did not employ allocation hiding; Hansen 2011 did not use participant blindness and had case shedding; Tofas 2021 and Theodorou 2016 did not mention whether participant blindness and evaluator blindness were applied. On that basis, significant heterogeneity of test results may be caused. And in Marzolini's study, two CRTS were present and compared with traditional resistance training. We used both methods as intervention groups to reach more logical conclusions.

It is generally known that cardiac rehabilitation in CHD patients has recently progressed rapidly. As PCI technology is leaping forward, the mortality of CHD patients has been effectively regulated, whereas the long‐term prognosis remains affected by adverse cardiovascular complications, low cardiac function, and other factors. How to improve cardiopulmonary endurance, quality of life, and long‐term prognosis of CHD patients via effective exercise is worth exploring in depth.

Circuit resistance training is a training measure based on the isometric contraction of muscles. On the whole, it refers to the patient's repeated training under moderate or low load strengths to gain more coordination and balance ability. Circuit resistance training can is capable of effectively improving the metabolic rate of trained muscle and the strength of skeletal muscle, as well as effectively improving the composition of the body, the metabolic rate and oxygen consumption of the body, and the output of the heart without increasing the peripheral resistance, and the cardiac function effectively. Accordingly, circuit resistance training acts as an effective exercise intervention to improve CHD patients. Exercise capacity can be recognized as a strong predictor of death; METs are a vital indicator for assessing exercise capacity and prognosis. The higher the exercise capacity, the lower the risk of myocardial ischemia will be. According to clinical studies, exercise can improve METs of patients with CHD, and every increase of 1MET can upregulate the survival rate by 12%.^19^

VO_2_ max is the amount of oxygen taken in and provided for oxidative use by tissues and cells per minute when the body's respiratory and circulatory systems function at their maximal level. It is currently considered the optimal index to assess cardiopulmonary function and exercise tolerance of patients.^20^

It was reported that circuit resistance training could significantly increase the VO_2_ peak in CHD patients compared with aerobic training.^7^, ^14^, ^17^ Other studies have shown that circuit resistance training does not improve the VO_2_ peak in patients with CHD.^16^, ^18^ Therefore, we conducted this study to assess the effects of circuit resistance training on the VO_2_ peak in CHD patients. According to the combined results of this meta‐analysis, compared with the aerobic training group, circuit resistance training insignificant impacts the VO_2_ peak in CHD patients. Possible reasons for the difference are presented below. First, the duration of exercise training in several studies was too short to detect the beneficial effects of cyclic resistance training on the VO_2_ peak in patients. Second, the sample size of the respective included study was excessively small. Third, the resistance training type is insufficiently comprehensive, exercise intensity is overly low, and exercise frequency is different. However, the VO_2_ peak was found to increase in the circuit resistance training group compared with aerobic training. Although the mentioned difference is insignificant, it has been reported that cardiac patients with a moderate advantage in the VO_2_ peak have significant benefits for physical function and long‐term outcomes.^21^

Arterial blood pressure is primarily determined by cardiac output and peripheral resistance. During aerobic exercise, multiple organs and skeletal muscles of the body are involved in the exercise. Cardiac output is improved with the increase in the exercise load, and the systolic blood pressure increases, while peripheral resistance decreases, and diastolic blood pressure remains unchanged or insignificantly decreases. After exercise, the systolic blood pressure decreased with the decrease in cardiac output. The blood supply of the myocardium is primarily completed at the diastolic stage, and the diastolic pressure directly affects the coronary artery perfusion pressure. On the whole, the resistance exercise is muscle contraction, largely causing the increase in the cardiac pressure load. Moderately increasing the diastolic pressure helps increase the myocardial perfusion, thereby effectively reducing the effect of aerobic exercise on the diastolic pressure. As indicated in the present meta‐analysis, circuit resistance training significantly impacts the systolic blood pressure compared with aerobic training, and the effect of circuit resistance training on diastolic blood pressure and mean arterial blood pressure could be more specifically discussed in the subsequent study and analysis.

In healthy populations, obesity and high body fat content are significantly correlated with metabolic disorders, morbidity, and mortality from cardiovascular events.^22^ Numerous studies evidenced that exercise training significantly impacts body weight and body composition.^23^ According to Pimenta et al.,^24^ aerobic combined resistance training could significantly reduce the body fat content of CHD patients but insignificantly impact the body weight and the BMI. This finding can be attributed to the subjects' modest increase in lean body mass and thus weight maintenance. Since the amount and distribution of fat combined with a reduction in skeletal muscle mass are correlated with an elevated cardiovascular risk, the serum total cholesterol level is positively correlated with the morbidity and mortality of CHD. Existing studies reported that aerobic exercise training and regular physical activity could moderately reduce the body mass and the fat content.^25^

Overall, CRT did not differ significantly from conventional resistance training in terms of patient benefit. However, CRT has the advantage of less load per session and lower training intensity. This makes CRT more beneficial for CHD patients, especially those with severe CHD or too weak physical conditions. Furthermore, CRT is obviously better than traditional resistance training in improving body fat in patients with CHD, which also makes CRT a better application prospect.

In this systematic review, circuit resistance training significantly reduced the BMI and the body fat content compared with aerobic training. Thus, it can effectively delay the progression of CHD and reduce mortality.

### 4.1 Local

In this systematic review, some studies have high inter‐heterogeneity and small sample size, so large‐sample and high‐quality RCT studies should be further conducted. The population and nations included in the respective study are different, and there may be regional bias. Differences exist in the frequency, intensity, duration, and type of exercise training adopted in various studies, thereby probably causing differences in the final measurement index results. The literature retrieval method has some limitations, and there may be omissions. This meta‐analysis includes only studies published in English, which can cause potential publication bias. A small number of included studies are insufficiently rigorous for the assignment hiding, the participant blindness, as well as the evaluator blindness.

## 5 CONCLUSIONS

In brief, although circuit resistance training fails to significantly impacts peak VO_2_ as compared with aerobic training independently, it helps reduce the body fat percentage and the BMI and more significantly facilitates the rehabilitation of CHD patients. For this reason, in exercise prescriptions for CHD patients, it is recommended that patients use circuit resistance training. The personalized exercise training plan of circuit resistance training can be further explored by complying with the specific situation of the patients. Patients should be given professional exercise guidance and supervision in exercise training, while the safety and quality of patient training should be ensured. Although there is not much significant difference between CRT and AT in the improvement of most indicators, CRT has advantages such as less load for patients.

## AUTHOR CONTRIBUTIONS

Shengnan Wang led the work and managed the team. Chunchun Wu formatted the manuscript and contributed to the data analysis. Rongsheng Bu and Yaoguo Wang contributed to the literature identification. Chaoxiang Xu and Youfang Chen helped in characteristic extraction and double‐checked the data used in meta‐analysis. Listing Che contacted with authors for some original research literatures. All authors participated in the study and have reviewed and approved the final manuscript.

## CONFLICT OF INTEREST

The authors declare no conflicts of interest.

## Supporting information

Supporting information.

## Data Availability

All the data used in the present meta‐analysis is available in PubMed (https://pubmed.ncbi.nlm.nih.gov/), Web of Science (https://www.webofscience.com/wos/woscc/basic-search), The Cochrane Library (https://www.cochranelibrary.com/search), Embase (https://www.embase.com), Clinical Trials (https://clinicaltrials.gov), and CNKI (www.cnki.net).
