# Supplementary material for: Rehabilitation effects of circuit resistance training in coronary heart disease patients: A systematic review and meta‐analysis
Source: Clin Cardiol. 2022 Jun 27;45(8):821–30. doi: 10.1002/clc.23855 (PMC9346966; doi:10.1002/clc.23855)
Supplement: Supplementary file 1 — Supporting information. [file CLC-45-821-s001.docx]

**Supplements**


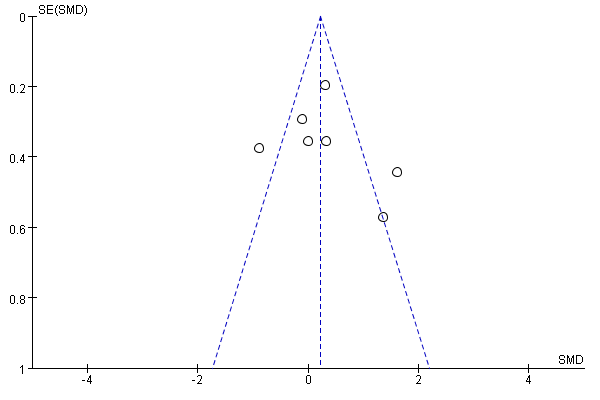
s Fig 1. VO2 peak Funnel plots for assessing publication bias.


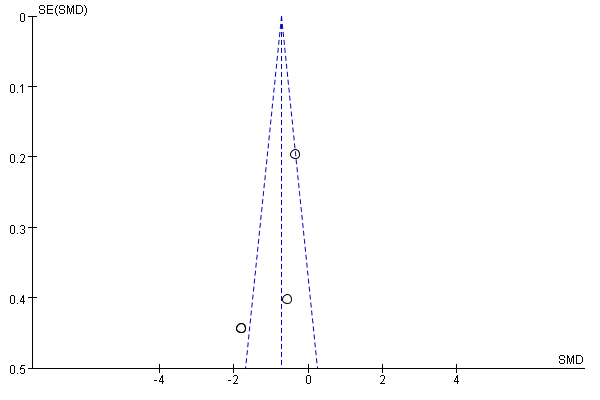
s Fig 2. BMI Funnel plots for assessing publication bias


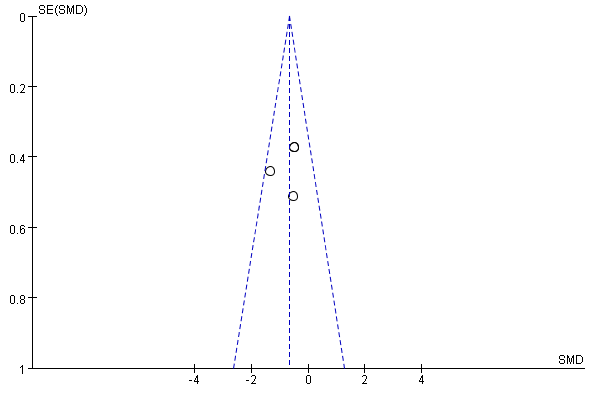
s Fig 3. (FM, in %) Funnel plots for assessing publication bias


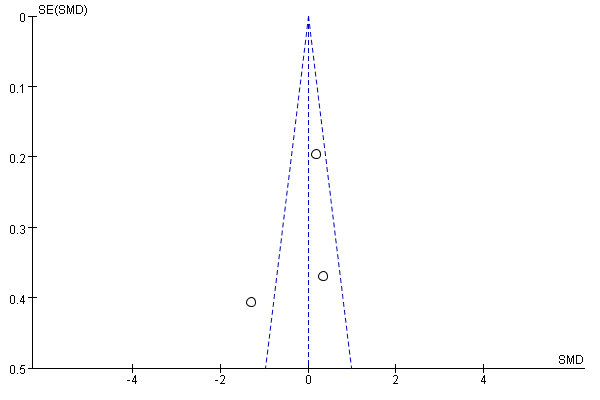
s Fig 4. SBP Funnel plots for assessing publication bias.


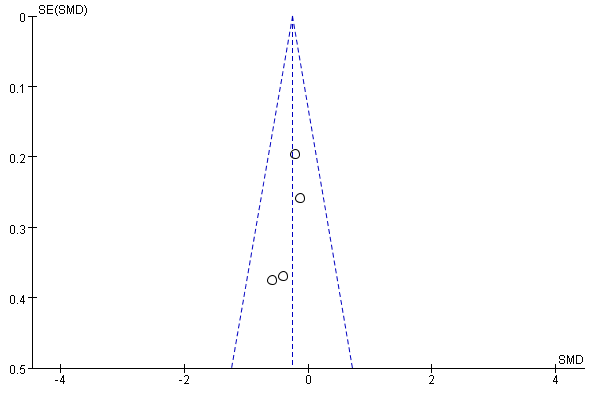
s Fig 5. CHO Funnel plots for assessing publication bias


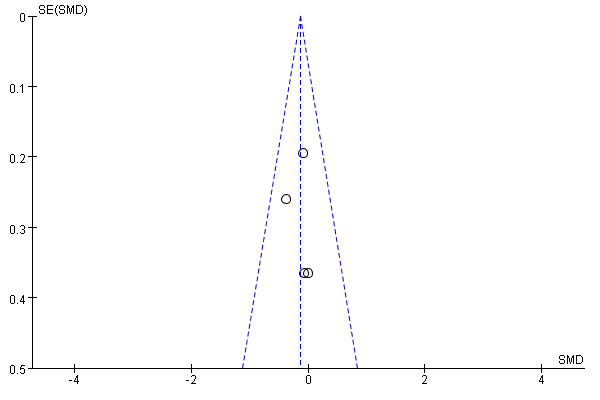
s Fig 6. TG Funnel plots for assessing publication bias.
